# Mitral valve transcriptome analysis in thirty-four age-matched Cavalier King Charles Spaniels with or without congestive heart failure caused by myxomatous mitral valve disease

**DOI:** 10.1007/s00335-023-10024-1

**Published:** 2023-11-08

**Authors:** Maria J. Reimann, Signe Cremer, Liselotte Christiansen, Emil Ibragimov, Fei Gao, Susanna Cirera, Merete Fredholm, Lisbeth H. Olsen, Peter Karlskov-Mortensen

**Affiliations:** 1 Preclinical Disease Biology, Department of Veterinary and Animal Sciences, Faculty of Health and Medical Sciences, University of Copenhagen, Frederiksberg, Denmark; 2 Animal Genetics and Breeding, Department of Veterinary and Animal Sciences, Faculty of Health and Medical Sciences, University of Copenhagen, Frederiksberg, Denmark; 3 Genome Analysis Laboratory of the Ministry of Agriculture, Agricultural Genomics Institute at Shenzhen, Chinese Academy of Agricultural Sciences, Shenzhen, China; 4 Comparative Pediatrics and Nutrition, Department of Veterinary and Animal Sciences, Faculty of Health and Medical Sciences, University of Copenhagen, Frederiksberg, Denmark

## Abstract

We here report the results of a mitral valve transcriptome study designed to identify genes and molecular pathways involved in development of congestive heart failure (CHF) following myxomatous mitral valve disease (MMVD) in dogs. The study is focused on a cohort of elderly age-matched dogs (*n* = 34, age ~ 10 years) from a single breed—Cavalier King Charles Spaniels (CKCS)—with a high incidence of MMVD. The cohort comprises 19 dogs (10♀, 9♂) without MMVD-associated CHF, and 15 dogs (6♀, 9♂) with CHF caused by MMVD; i.e., we compare gene expression in breed and age-matched groups of dogs, which only differ with respect to CHF status. We identify 56 genes, which are differentially expressed between the two groups. In this list of genes, we confirm an enrichment of genes related to the TNFβ-signaling pathway, extracellular matrix organization, vascular development, and endothelium damage, which also have been identified in previous studies. However, the genes with the greatest difference in expression between the two groups are *CNTN3* and *MYH1*. Both genes encode proteins, which are predicted to have an effect on the contractile activity of myocardial cells, which in turn may have an effect on valvular performance and hemodynamics across the mitral valve. This may result in shear forces with impact on MMVD progression.

**Supplementary Information:**

The online version contains supplementary material available at 10.1007/s00335-023-10024-1.

## Introduction

Myxomatous mitral valve disease (MMVD) is the most common heart disease in dogs. It is especially common in small size dog breeds, but the Cavalier King Charles Spaniel (CKCS) stands out as a breed with an extraordinarily high prevalence of this disease (Darke 1987; Häggström et al. 1992).

MMVD has been intensively studied over the last decades, and several excellent reviews have been published (e.g., Aupperle and Disatian 2012; Borgarelli and Buchanan 2012; Borgarelli and Haggstrom 2010; Burchell and Schoeman 2014; Fox 2012; O’Brien et al. 2021). The disease is characterized by morphological changes in the mitral valve leaflets, chordae tendineae, and chordal-papillary muscle. The changes include elongation and thickening of the valves in combination with derangement of connective tissue, reduction in connective tissue density, damage to the basement membrane and endothelium, damage to the valve collagen matrix and accumulation of immature collagens, laminin, proteoglycans, and glycosaminoglycans in the extracellular matrix (ECM) of the mitral valves (Aupperle et al. 2009a; Buchanan 1977; Corcoran et al. 2004; Falk et al. 2010; Hadian et al. 2010; Han et al. 2010, 2013a, 2013b). The morphological changes result in inadequate biomechanical properties of the heart valves, insufficient closure, and subsequent regurgitation of blood from the left ventricle to the left atrium (Sargent et al. 2015). In severe cases, consequences of MMVD include congestive heart failure (CHF) resulting in reduced expected lifespan and reduced life quality for both dog (Boswood et al. 2018; Haggstrom et al. 2013) and owner (Clements et al. 2003). Lifelong treatment is usually required (e.g., Keene et al. 2019). Alternatively, heart valve surgery is a possibility (e.g., Matsuura et al. 2022).

It has been demonstrated that genetics play an important role in MMVD (Olsen et al. 1999; Stern et al. 2015; Swenson et al. 1996), and selective breeding can reduce MMVD prevalence in CKCS (Birkegard et al. 2015). Numerous studies have been performed to identify and elucidate the genetic and molecular mechanisms underlying MMVD. This includes genome-wide association studies, whole genome-sequencing studies, proteome analysis, micro-RNA studies, gene expression/ transcriptome studies, and other genomic studies. While previous gene expression studies have included several dog breeds of different age and multiple stages of MMVD, the present study focuses exclusively on CKCS and, more specifically, on two large age-matched groups of CKCS where one group comprises dogs without CHF and another group of dogs have CHF caused by MMVD. Hence, the objective of the present study is to compare gene expression in mitral valves from ~ 10-year-old CKCS dogs with MMVD, which has progressed into CHF, to gene expression in other dogs of a similar age of the same breed.

## Materials and methods

### Animals and sample collection

**Fig. 1:**
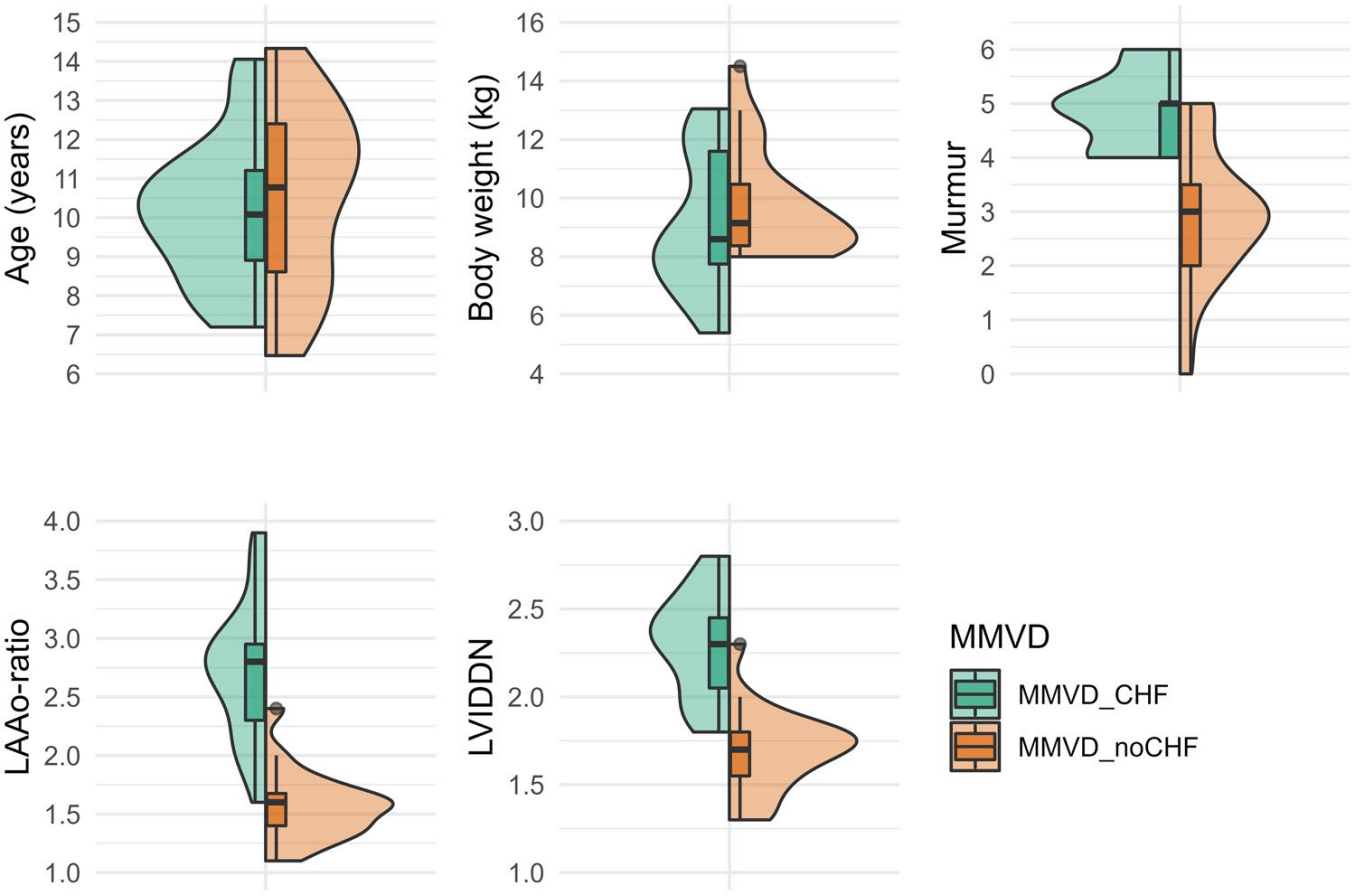
Split-violin plots and box plots illustrating descriptive statistics and key diagnostic parameters for Cavalier King Charles Spaniel dogs without CHF (*n* = 19) and MMVD affected Cavalier King Charles Spaniel dogs with CHF (*n* = 15). LVIDDN = left ventricular end-diastolic diameter normalized for body weight. LAAo ratio = the ratio of diameters for the left atrium and aorta. Based on the box plots, two dogs without CHF were identified as outliers (gray dots): One with regard to body weight and another dog with regard to LAAo ratio and LVIDDN. Both were kept in the dataset based on an overall evaluation of their status

Privately owned CKCS with no MMVD or different stages of MMVD were recruited at time for elective euthanasia at Department of Veterinary and Animal Sciences, University of Copenhagen. The dogs were collected from August 2008 to August 2020. The study was approved by the Danish Animal Experiments Inspectorate (licenses no. 2006/561-1145, 2011/561-71 and 2016-15-0201-01074). Dogs with cardiac disease other than MMVD were excluded. Some of the dogs have previously been included in other studies with other research aims (contact the corresponding author for a complete list of studies). Prior to euthanasia and upon written-owner consent, all dogs underwent a clinical examination using a standardized protocol including owner interview, physical examination, auscultation, and echocardiography as previously described (Reimann et al. 2021). Mitral regurgitation murmur intensity was graded on a scale of 1–6 (Gompf 1988). The diagnosis of MMVD was based on auscultation, echocardiography, and the presence of clinical signs of CHF due to MMVD, according to the American College of Veterinary Internal Medicine (ACVIM) MMVD classification guidelines (Keene et al. 2019). Dogs examined before 2019 were retrospectively classified on the basis of the measurements obtained at the time of their examination. Echocardiographic assessment and ACVIM staging were performed as described elsewhere (Reimann et al. 2021). All dogs, included in the study, were approximately 10 years old (Table 1, Fig. 1). The dogs were divided in two groups based on symptoms of CHF. One group contained one dog with no identifiable structural disorder of the heart (ACVIM stage A) plus dogs with MMVD at ACVIM stages B1 and B2. In this study, we will collectively term this group of dogs ‘10y-noCHF.’ The other group of dogs, which we in this study will term ‘10y-MMVD-CHF,’ were diagnosed with CHF due to MMVD (ACVIM stage C). CHF diagnosis was based on a history of MMVD, previous or current clinical signs of CHF (e.g., cough, dyspnea, tachypnea, nocturnal restlessness, and exercise intolerance), echocardiographic changes compatible with severe MMVD, and response to diuretic treatment. Dogs were euthanized using pentobarbitale (200–400 mg/kg or until effect, IV) after sedation and pain relief with butorphanol (0.1 mg/kg, IM) and dexmedetomidine (0.02 mg/kg, IM). The heart was collected, and the mitral valves were excised within 60 min after euthanasia and stored in RNAlater (Merck KGaA, Darmstadt, Germany).

**Table 1:** Descriptive statistics for dogs classified as 10y-noCHF (*n* = 19) and 10y-MMVD-CHF (*n* = 15)

	Mean		Median		sd		Min		Max	
	10y-noCHF	10y-MMVD-CHF	10y-noCHF	10y-MMVD-CHF	10y-noCHF	10y-MMVD-CHF	10y-noCHF	10y-MMVD-CHF	10y-noCHF	10y-MMVD-CHF
Age (years)	10.6	10.2	10.8	10.1	2.35	1.92	6	7	14	14
Body weight (kg)	9.82	9.14	9.15	8.6	1.87	2.43	8.1	5.4	14.5	13.05
Murmur	2.9	4.9	3	5	1.3	0.7	0	4	5	6
LAAo ratio	1.56	2.70	1.56	2.80	0.30	0.60	1.1	1.6	2.4	3.9
LVIDDN	1.70	2.29	1.7	2.3	0.26	0.30	1.3	1.8	2.3	2.8

Descriptive statistics for ~ 10 years old CKCS without CHF (10y-noCHF) and ~ 10-year- old MMVD affected dogs where MMVD has progressed into a stage of CHF (10y-MMVD-CHF)

*sd* standard deviation, *Min* minimum, *Max* maximum, *LAAo ratio* the ratio of diameters for the left atrium and aorta, LAAo ratio was missing for one dog with asymptomatic MMVD. *LVIDDN* Left ventricular end-diastolic diameter normalized for body weight

Three dogs (two B2 and one C) underwent the full clinical examinations, auscultation, and echocardiography 4 months before euthanasia. None of the B2 stage dogs had developed clinical signs that indicated CHF on the day of euthanization. Tissue collection was delayed for one dog (in RNAlater 90 min after euthanasia). This had no observable effect on RNA quality or gene expression results.

### RNA isolation and sequencing

RNA was isolated from mitral valves using the Qiagen RNeasy Fibrous Tissue Mini Kit (Qiagen, Hilden, Germany), according to the manufacturer’s instruction including DNase treatment. RNA quantity was measured using a NanoDrop 1000 spectrophotometer (Thermo Fisher Scientific, Waltham, Massachusetts, USA) and RNA quality, expressed as an RNA Integrity Number (RIN), was determined for each sample using a 2100 Bioanalyzer (Agilent Technologies, Santa Clara, California, USA). Subsequently, mRNA was purified from total RNA using poly-T oligo-attached magnetic beads. Fragmentation was carried out using divalent cations under elevated temperature in First-Strand Synthesis Reaction Buffer (5X). First-strand cDNA was synthesized using random hexamer primer and M-MuLV Reverse Transcriptase, and then RNaseH was used to degrade the RNA. Second-strand cDNA synthesis was subsequently performed using DNA polymerase I and dNTP. Remaining overhangs were converted into blunt ends via exonuclease/polymerase activities. After adenylation of 3’ ends of DNA fragments, adaptors with hairpin loop structure were ligated to prepare for hybridization. In order to select cDNA fragments of preferentially 370 ~ 420 bp in length, the library fragments were purified with AMPure XP system (Beckman Coulter, Beverly, USA). After PCR amplification, the PCR product was purified by AMPure XP beads, and the sequencing library was finally obtained. Libraries were constructed at E-GENE, Shenzhen, China and sequenced by an Illumina NovaSeq 6000 platform at Novogene, Beijing, China. Paired-end sequencing reads of 150 bp were generated.

RNA sequence quality was ascertained using FastQC (Andrews 2010), and reads were mapped to the canFam4/UU_Cfam_GSD_1.0 dog genome assembly using the RNA sequence aligner STAR (Dobin et al. 2013). Subsequently, a feature annotation file was compiled based on the canFam4 NCBI-RefSeq annotation. This set of features was augmented by a set of putative genes extracted among canFam4 aligned human XenoRefSeq genes from the UCSC genome browser repository. This step captured the presence of protein-coding genes, which may not have been properly annotated in the canine genome. Mapped RNA sequence reads were concomitantly assigned to genomic features and counted using featureCounts (Liao et al. 2014). Genes with low expression, i.e., features with a sum of less than 100 counts across all samples, were filtered out and excluded from further analysis.

### Analyses of gene expression

The BioLayout-3.4 network analysis tool (Theocharidis et al. 2009) was used to cluster samples based on similarity of overall gene expression pattern and visualize the results in a three-dimensional network where nodes represent samples and the distance in space between nodes represents the correlation in gene expression between two samples. When cases and controls are marked up in the network, any clustering according to CHF status will be observable. The tool was used to calculate Pearson correlation coefficients based on gene expression data, and the advanced graph layout Fruchterman–Rheingold algorithm was used to visualize the results. In order to minimize noise and to emphasize biologically meaningful results, very abundant features (i.e., essential household genes), with over 10,000 reads per feature per sample on average, were filtered out before the BioLayout analysis. A Pearson correlation coefficient cut-off of 0.93 was used for clustering. This was the highest cut-off that allowed a combined cluster of all samples.

An analysis of differences in gene expression between dogs classified as 10y-noCHF and dogs classified as 10y-MMVD-CHF was performed using the R package DESeq2 (Love et al. 2014). Analyses were performed on the raw un-normalized feature count data in accordance with the DESeq2 documentation and guidelines. Sex was included as a fixed effect. A volcano plot illustrating the results was created using the R package ggplot2 (Wickham 2016). A Benjamini–Hochberg false discovery rate (FDR) threshold of 0.1 was used to identify significant DE genes.

### STRING network analysis

A STRING network and enrichment analysis was performed using Cytoscape (Jensen et al. 2009; Shannon et al. 2003). The analysis was performed using gene names for all DE genes with a FDR < 0.1. Two analyses were performed, one using the STRING default-confidence score of 0.4 and one using a stringent-confidence score of 0.9. Furthermore, up to 10 additional interactors were allowed in both analyses. The default-confidence network provided an overview of the functional networks of which the identified DE genes play a role. The stringent-confidence network enabled identification of the functions for which the dataset provided the strongest evidence. Inclusion of additional interactors allowed STRING to build a network even though specific components (genes) could be missing in the dataset.

## Results

Thirty-four dogs were included in the study. Nineteen dogs (10 females and 9 males) were classified as 10y-noCHF. These included one dog with ACVIM stage A, 10 dogs with ACVIM stage B1, and eight dogs with ACVIM stage B2. Furthermore, 15 dogs (6 females and 9 males) were diagnosed with CHF due to MMVD indicated by ACVIM stage C. Age and body weight did not differ significantly between the two groups of dogs (Table 1, Fig. 1).

RNA isolation resulted in 34 samples with a RIN number > 8.6 (average 9.23, sd = 0.37) and sequencing resulted in 22.4—35.6 M reads per sample with an average of 27.7 M reads per sample remaining after the quality check.

**Fig. 2:**
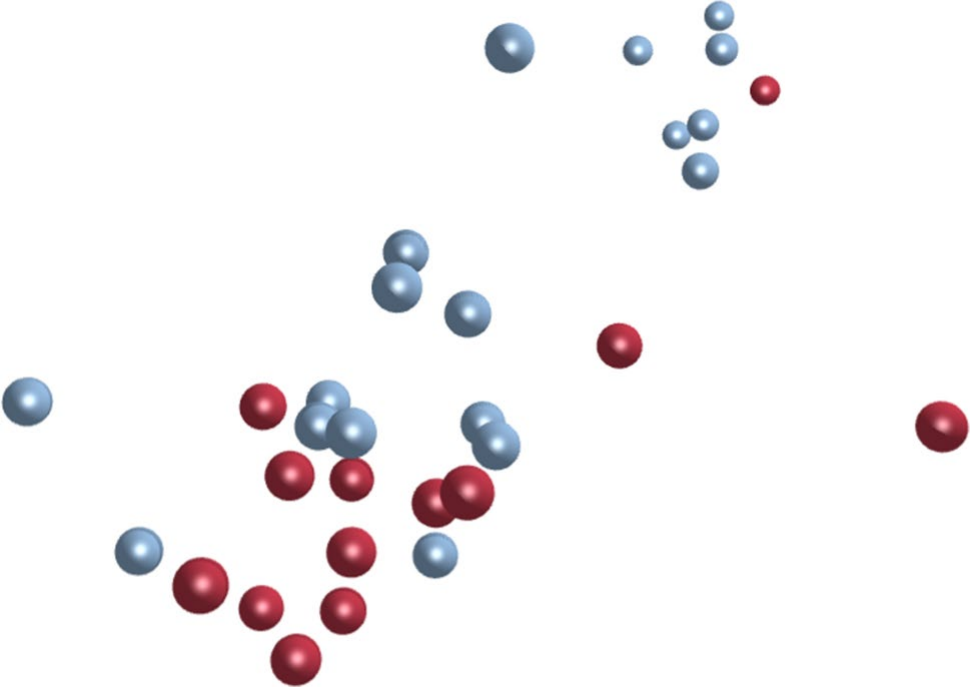
Three-dimensional illustration of the results of the BioLayout gene expression correlation analysis. Blue = Dogs classified as 10y-noCHF; Red = Dogs classified as 10y-MMVD-CHF. The distances between dots corresponds to the correlations in overall gene expression between individuals

The result of the BioLayout gene expression correlation analysis is illustrated in a three-dimensional sample-to-sample-weighted network graph as shown in Fig. 2. Dogs in the 10y-noCHF group are marked with blue spheres, and dogs in the 10y-MMVD-CHF group are marked using red spheres. A clear clustering of 10y-MMVD-CHF dogs is observed. This indicates that the advanced stage of MMVD, at which CHF had developed, was associated with a distinct gene expression pattern. However, it is also evident that several dogs in the 10y-noCHF group had expression patterns that overall resembled the pattern observed in MMVD affected dogs with CHF. This reflects the heterogeneous nature of the 10y-noCHF group where several dogs have severe MMVD although they have not developed CHF.

**Fig. 3:**
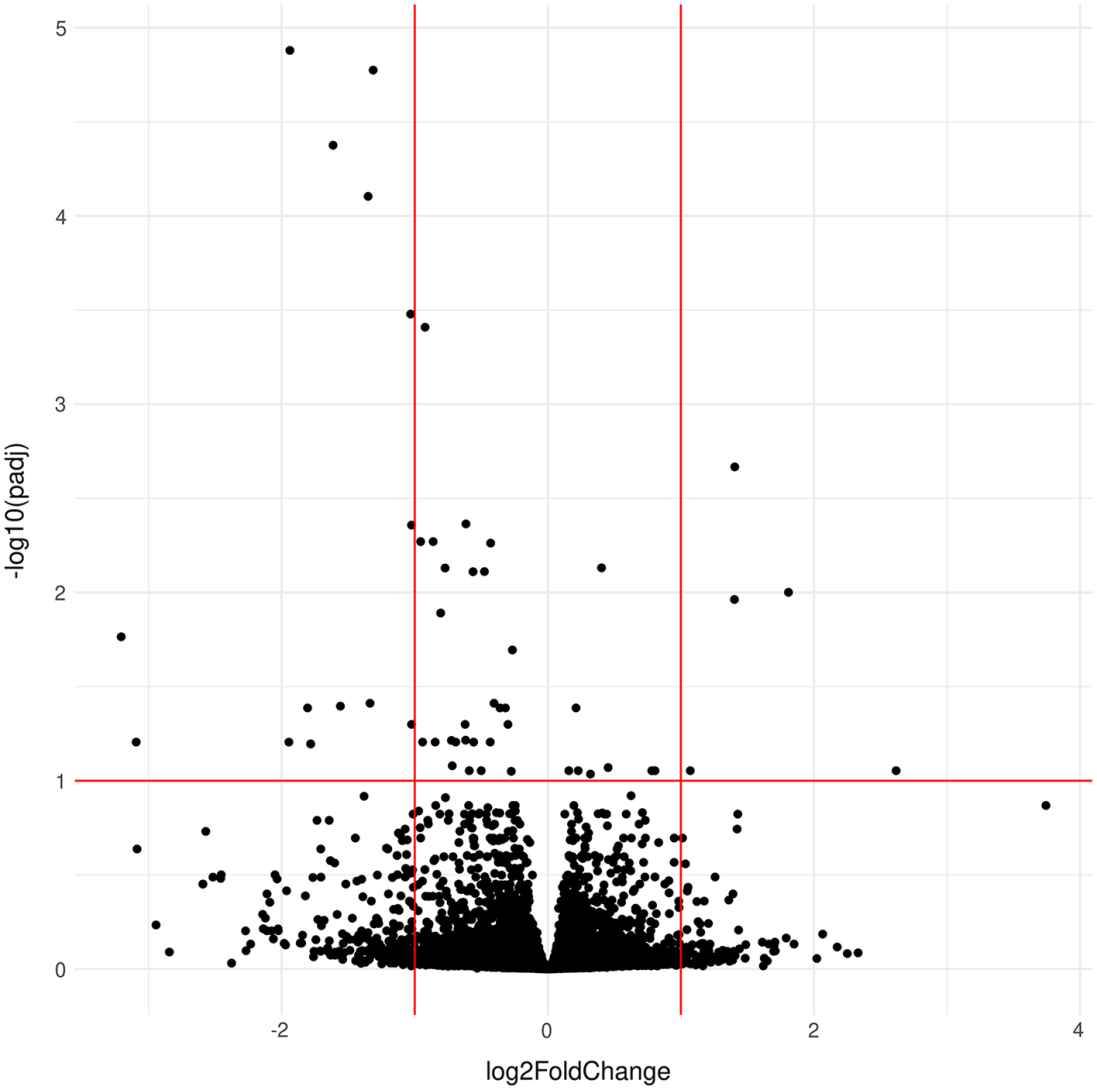
Volcano plot of DESeq2 analysis results. The log2FoldChange is on the X-axis and the negative logarithm of the false discovery rate (FDR = p-adj) is on the Y-axis. The FDR threshold of 0.1 is indicated by a horizontal line. Vertical lines indicate log2FoldChanges of − 1 and + 1

A more detailed gene-by-gene analysis of differences in gene expression between dogs classified as 10y-noCHF and dogs classified as 10y-MMVD-CHF was performed using the DESeq2 software package. We used a significance threshold of 0.1 for the p-adj value calculated by DESeq2. This value is a Benjamini–Hochberg FDR, which effectively corrects for multiple testing. The analysis revealed a set of 56 genes, which were significantly differentially expressed between the two groups (Table 2, Fig. 3).

**Table 2:** Differentially expressed genes and results of the DESeq2 analysis

Gene	baseMean	log2FoldChange	lfcSE	pvalue	FDR
*CNTN3*	43.36234	2.61744	0.720429	0.00028	0.088436
*NPPC*	442.813	1.80895	0.411836	1.12E-05	0.00998
*NPTX1*	170.2808	1.40521	0.287143	9.89E-07	0.002153
*VWF*	8267.843	1.4027	0.321461	1.28E-05	0.010899
*ASS1*	330.1307	1.07067	0.292604	0.000253	0.088436
*ITIH4-AS1*	1330.625	0.80563	0.22078	0.000263	0.088436
*RDH10*	1826.389	0.78186	0.214862	0.000274	0.088436
*MOSPD2*	7483.611	0.45354	0.123139	0.00023	0.085134
*MAOA*	9924.007	0.35148	0.0725	1.25E-06	0.002442
*GALNT10*	5876.648	0.32077	0.088751	0.000301	0.092185
*MXI1*	3437.44	0.22864	0.06254	0.000256	0.088436
*BAG4*	1162.375	0.21209	0.053333	6.99E-05	0.041049
*RBM45*	745.1913	0.15857	0.043351	0.000254	0.088436
*PDLIM1*	26,336.33	− 0.265809	0.063434	2.79E-05	0.020205
*LMNA*	24,307.17	− 0.27434	0.075638	0.000287	0.089134
*XPC*	1711.009	− 0.299047	0.076738	9.74E-05	0.050202
*AIF1L*	2647.525	− 0.317861	0.079938	7.00E-05	0.041049
*COBLL1*	2335.624	− 0.358934	0.090523	7.34E-05	0.041049
*DUSP10*	581.7944	− 0.403967	0.100407	5.74E-05	0.038756
*CRIP1*	9012.843	− 0.429343	0.093299	4.19E-06	0.005469
*ZNF571*	242.5365	− 0.432631	0.114389	0.000156	0.06233
*TNXA*	18,968.13	− 0.476062	0.106024	7.12E-06	0.007744
*ADGRG2*	473.3885	− 0.500322	0.137694	0.00028	0.088436
*COL18A1*	3645.546	− 0.557163	0.147208	0.000154	0.06233
*CYP21A1P*	3409.611	− 0.561532	0.125913	8.21E-06	0.007758
*CYP21A2*	3409.611	− 0.561532	0.125913	8.21E-06	0.007758
*GPRC5A*	659.3588	− 0.589977	0.162223	0.000276	0.088436
*C17orf58*	720.3526	− 0.614794	0.130416	2.43E-06	0.004323
*PTPRD*	1191.211	− 0.617355	0.16062	0.000121	0.060893
*COL4A6*	712.5039	− 0.621122	0.159258	9.61E-05	0.050202
*ADAMTS19*	1484.077	− 0.69159	0.1819	0.000144	0.06233
*TSLP*	94.83645	− 0.717858	0.194356	0.000221	0.083298
*DNAH2*	52.46112	− 0.724051	0.188928	0.000127	0.061083
*PRSS22*	92.07796	− 0.771723	0.171058	6.44E-06	0.007417
*ATF3*	909.1752	− 0.80483	0.186398	1.58E-05	0.012859
*SERPINE1*	9835.401	− 0.846452	0.223576	0.000153	0.06233
*IL1R2*	371.619	− 0.861579	0.186134	3.68E-06	0.005368
*TPPP3*	2813.976	− 0.922035	0.175911	1.59E-07	0.00039
*COL17A1*	19.7643	− 0.931678	0.235232	3.960674	7.47E-05
*KLK4*	104.6258	− 0.940408	0.248693	0.000156	0.06233
*ADAMTS6*	1248.118	− 0.95533	0.206781	3.84E-06	0.005368
*MLXIPL*	146.4413	− 1.023708	0.262493	9.62E-05	0.050202
*PDZRN4*	126.4177	− 1.023815	0.218143	2.69E-06	0.004387
*AKNAD1*	47.59294	− 1.030386	0.194578	1.19E-07	0.000332
*BMPER*	474.4934	− 1.312184	0.2203	2.58E-09	1.68E-05
*ALDH1A2*	104.5354	− 1.335844	0.331976	5.72E-05	0.038756
*TNFSF4*	252.9707	− 1.349945	0.240581	2.01E-08	7.87E-05
*SCN1A*	82.82639	− 1.558178	0.388886	6.16E-05	0.040151
*FOSB*	579.1971	− 1.613517	0.280305	8.60E-09	4.21E-05
*SSUH2*	36.48473	− 1.781245	0.473041	0.000166	0.063824
*NTS*	57.83466	− 1.781337	0.469854	0.00015	0.06233
*EREG*	158.5861	− 1.804959	0.455037	7.29E-05	0.041049
*LAMC2*	552.8708	− 1.937688	0.316755	9.52E-10	1.32E-05
*MYHAS*	30.95253	− 1.94536	0.510493	0.000139	0.06233
*LOC102724058*	4.398863	− 3.092977	0.81132	0.000138	0.06233
*MYH1*	14.92086	− 3.205454	0.756854	2.28E-05	0.017198

Results of the DESeq2 analysis; baseMean = Average of normalized feature counts across all samples; lfcSE = standard error of log2FoldChange; pvalue = unadjusted *p* value; FDR = Benjamini–Hochberg false discovery rate

Compared to dogs in the 10y-noCHF group, five genes were up-regulated with a log2FoldChange > 1 and fifteen were down-regulated with a log2FoldChange <  − 1 in CKCS with MMVD-associated CHF. Among these DE genes, two genes, *MYH1* and LOC102724058, were down-regulated with a log2FoldChange <  − 3. The most up-regulated gene was *CNTN3* with a log2FoldChange of 2.62.

**Fig. 4:**
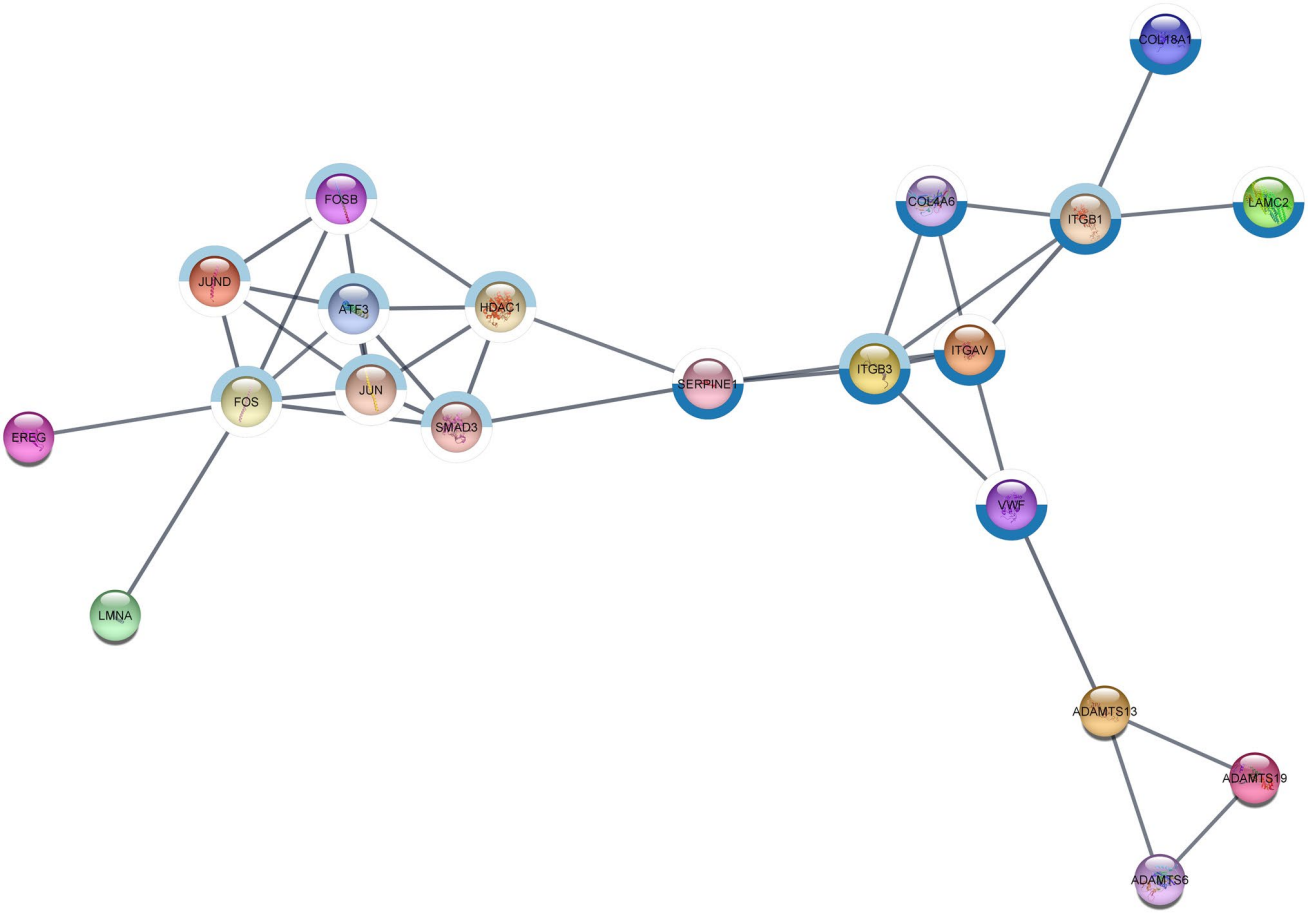
High confidence large functional network constructed using STRING. Marked with light blue: Genes involved in TGFβ signaling. Marked with dark blue: Genes involved in extracellular matrix organization

**Fig. 5:**
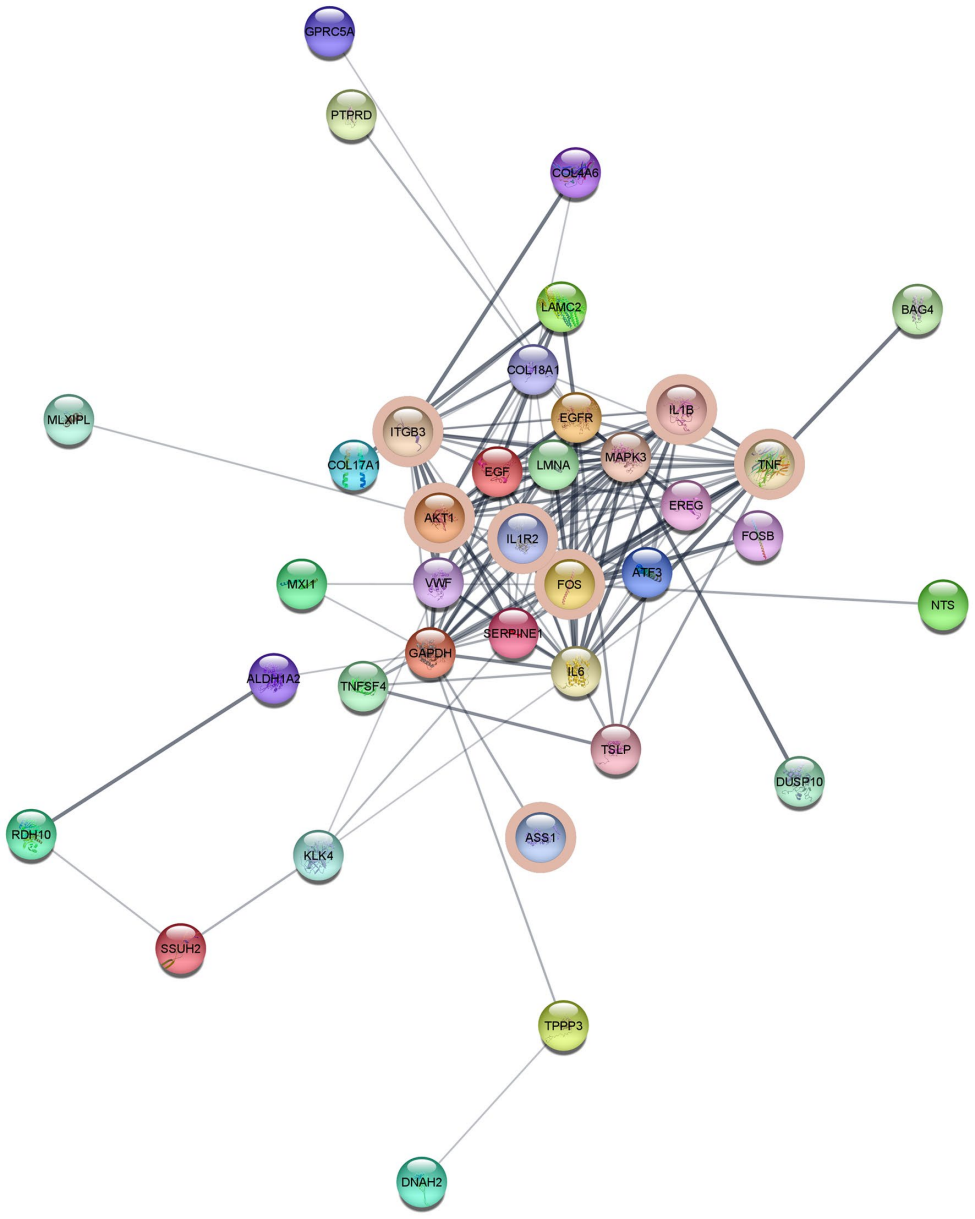
Default confidence large functional network constructed using STRING. Genes associated with the KEGG pathway “Fluid shear stress and atherosclerosis” are marked with pink circles

A STRING network analysis for significant DE genes allowing for ten additional interactors and a stringent-confidence level of 0.9 revealed a functional network incorporating 20 genes (13 DE genes plus 7 additional interactors) (Fig. 4) and a small network of three genes (2 DE genes, *ALDH1A2*, *RDH10* plus 1 additional interactor, *CYP26A1*). A functional enrichment analysis of the large network revealed a predominance of genes involved in TGFβ signaling and ECM organization, whereas the three genes in the small network all were involved in retinol metabolism. In addition, genes in the large network were involved in focal adhesion, laminin interaction, circulatory system development and more (see Supplementary Table S1). We, furthermore, used the full set of DE genes to build networks using the STRING default-confidence level of 0.4. This resulted in a network comprising 37 genes of which 27 were DE genes, and the remaining 10 genes were additional interactors (Fig. 5). A functional enrichment analysis on the default-confidence network revealed significant enrichment of genes involved in, e.g., ‘regulation of cell population proliferation,’ ‘estrogen-dependent nuclear events downstream to ESR-membrane signaling,’ and ‘fluid shear stress and atherosclerosis’ (Supplementary Table S2).

Four of the 13 DE genes in the high-confidence large network had an observed |Log2FoldChange|< − 1. Ten of the 27 DE genes in the default-confidence large network had a |Log2FoldChange|< − 1. It is worth noticing that the genes with the greatest difference in gene expression between CKCS in the 10y-noCHF and the 10y-MMVD-CHF group (|Log2FoldChange|< − 2) were not part of the identified networks.

## Discussion

We here report the results of a mitral valve transcriptome analysis performed in a cohort of 34 age-matched CKCS dogs with or without CHF caused by their MMVD.

We identified 56 genes, which were differentially expressed between the two groups. Among these, we identified genes, which previously have been associated with heart disease, and gene networks, which previously have been associated with MMVD development. Furthermore, we identified a small number of genes of importance for sarcomere assembly and function, which we hypothesize, may have a close connection with the genetic causes of MMVD.

It should be noted that we did not strictly compare healthy and affected dogs. All dogs (except one) had MMVD, but for one group, the disease had progressed into CHF whereas for the other group, it had not, even though all dogs had approximately the same age. We classified these two groups as cases and controls, respectively. The majority of the control dogs (10y-noCHF) had a clear heart murmur due to mitral regurgitation, and they were classified as ACVIM stages B1 or B2. The cases (10y-MMVD-CHF), on the other hand, were classified as ACVIM stage C due to their symptoms of CHF. Age of dogs in both groups was around 10 years, with a slightly higher average age in the 10y-noCHF group. It would have been preferable to have a control group of CKCS dogs without any sign of MMVD. Unfortunately, as we aimed to perform this study on age-matched groups of dogs, such dogs were not available. The majority of elderly CKCS dogs have some degree of murmur due to regurgitation (Beardow and Buchanan 1993).

Previous MMVD-focused transcriptome studies have typically been performed on 3–6 MMVD cases and a similar number of controls. Most often there has been an uneven distribution of breeds between cases and controls and a significant difference in age between the two groups (Li et al. 2015; Lu et al. 2015; Markby et al. 2017, 2020a, 2020b; Oyama and Chittur 2006; Zheng et al. 2009). Significant strengths in the present study are that we included more individuals and that the two groups of dogs only differed in one parameter, i.e., their CHF status. Thus, the number of possible confounding factors was smaller compared to previous studies.

Compared to several of the above-mentioned transcriptome studies, the present study found a relatively low number of DE genes. This was probably due to the close similarity between the case and control groups and the absolute minimization of confounding factors in the present study. Furthermore, the larger sample size improved the power to avoid spurious differences in feature counts between the two groups, i.e., it reduced the risk of false-positive results in the DE analysis. The benefit of this is that the observed differences in gene expression can more reliably be assigned to case/control status.

In the functional enrichment analysis, we identified some of the same pathways as have been identified in previous studies. This included the TNFβ-signaling pathway, pathways related to ECM organization, and pathways related to vascular development, endothelium damage, and metallopeptidases (Aupperle et al. 2009b; Li et al. 2015; Lu et al. 2015; Markby et al. 2020a, 2020b; Moesgaard et al. 2014; Oyama and Chittur 2006; Zheng et al. 2009).

Among DE genes detected in the present study, *CRIP1* and *SERPINE1* were also identified as DE genes by Markby et al. (2020b). In a more detailed comparison of our results with this study, we noticed that many of the DE genes detected in the two studies were members of the same gene families. For example, several ADAMTS variants, collagen genes, laminins, and different myosin genes were detected in both studies.

Some of the DE genes identified in the present study have previously been directly linked to cardiac diseases including valve disease. *ADAMTS19* has been associated with progressive non-syndromic heart valve disease (Wünnemann et al. 2020). *ALDH1A2* has been linked to human congenital heart disease (Pavan et al. 2009). *BMPER* regulates cardiomyocyte size and changes in the mitral valve have been detected in *BMPER* knockout mice (Willis et al. 2013). *COL17A1* has been linked to mitral regurgitation and mitral valve prolapse in humans (Uysal et al. 2022). *CRIP1* expression has been associated with cardiac hypertrophy and an increased risk of stroke (Zeller et al. 2017). A partial deletion of *CYP21A2* has been found in patients with mitral valve prolapse (Chen et al. 2009). *MAOA* can play a role in the pathogenesis of heart failure (Kaludercic et al. 2011). *MYH1* is one of many genes important for the morphogenesis of the heart (England and Loughna 2013; Henderson et al. 2017), and *MYHAS* encodes an antisense RNA, which regulates expression of myosin heavy chain genes including *MYH1* (Haddad et al. 2003).

We primarily found a number of genes down-regulated in dogs with MMVD-induced CHF and only a few genes up-regulated in this group. Many of the down-regulated genes play a central role in mechanisms, which can be considered beneficial for heart maintenance. Hence, a relevant question is, why these genes appear to be down-regulated in dogs that have developed heart failure and what role, if any, their down-regulation plays in the development of CHF?

Among the DE genes identified in the present study, *ALDH1A2* and *RDH10* formed a small high-confidence functional network together with the additional interactor *CYP26A1*. The network was closely connected with retinoic acid (RA) biosynthesis, i.e., the oxidization of retinol (vitamin A) to retinaldehyde and the subsequent irreversible conversion of retinaldehyde to RA. RDH10 is the primary enzyme responsible for the first step in this reaction (Farjo et al. 2011), while the later step is catalyzed by retinaldehyde dehydrogenases (RALDHs) among which, the aldehyde dehydrogenase 1A2 (ALDH1A2) is the major form involved in cardiac development (Moss et al. 1998; Niederreither et al. 1997). A number of variations in *ALDH1A2* have been described in human patients with congenital heart disease (CHD), but none of them have been confirmed as significant modifiers of the risk of CHD in humans (Pavan et al. 2009). Genes involved in RA biosynthesis have previously been associated with MMVD in dogs. A retinoic acid receptor responder (*RARRES3*) was associated with MMVD in a microarray gene expression study performed in 10 CKCS dogs with MMVD, Whitney grade ≥ 3 and 6 dogs without signs of MMVD (Lu et al. 2015). Furthermore, RA signaling and the *ALDH1A2* gene have been linked to cardiac repair mechanisms in mice (Da Silva et al. 2021). Hence, we suggest that the observed changes in expression of *ALDH1A2* and *RDH10* may be a compensatory reaction to MMVD rather than a cause of disease. Why some dogs had an appropriate up-regulation of these genes and why some did not, needs to be investigated further.

Overall, it must be expected that compensatory mechanisms to disease, including MMVD, are established and managed in an orchestrated way, which in a transcriptome analysis will appear as networks of functionally related genes expressed in a coordinated manner. Hence, it is not surprising that many of the genes in the identified larger networks are involved in mechanisms such as TGFβ-signaling and ECM organization. Abundance of myxomatous effector proteins has previously been shown to increase in response to increased tensile strain on the heart valves (Lacerda et al. 2012; Orton et al. 2012). TGFβ signaling and disturbances in ECM organization have also been suggested as primary causes of MMVD (reviewed by Tang et al. 2022). However, the present results, i.e., the apparent well-orchestrated expression of genes related to these pathways, encourage us to suggest that these pathways were up-regulated or uphold as a well-regulated compensatory mechanism to MMVD in ~ 10-year-old dogs without CHF.

On the other hand, the genes with the greatest difference in expression between 10y-noCHF and 10y-MMVD-CHF dogs were three genes, *MYH1*, LOC102724058, and *CNTN3*, which were not part of the identified gene networks, i.e., they were not part of an orchestrated response to disease.

Of these three genes, LOC102724058 is a human gene that aligns to a 17 kb region of canine chromosome 36 with 91% identity. It was one of the features that were identified by augmenting the canine reference annotation with homologous human genes (see Material & Methods section). The gene is a long non-coding RNA gene with unknown function. The other two genes, *CNTN3* and *MYH1*, encodes contactin 3 and myosin heavy chain 1, respectively. Contactin 3 has among other things been associated with heart rate recovery after exercise (Verweij et al. 2018). *MYH1* expression has been found to be significantly up-regulated in mice with cardiomyopathy (Szema et al. 2013). Both of these genes relate to the contractile activity of the heart, the coordination of this activity, and consequently the hemodynamics across the mitral valves. This may explain the observed DE of genes in the default-confidence network related to ‘fluid shear stress and vascular changes,’ which, furthermore, corroborate the previously observed arteriosclerotic changes in dogs with MMVD (Falk et al. 2006). Comparatively, a vast number of different human myopathies including cardiomyopathies are caused by mutations in one of the many cytoskeletal sarcomeric proteins, of which *MYH1* is one (reviewed by Henderson et al. 2017). Mitral valve function relies on proper biomechanical performance of several structural components including the left atrial, ventricular, and papillary myocardium (Fox 2012; Richards et al. 2012; Schoen 2008). Hence, a disturbance in the heart’s contractile activity due to suboptimal coordination of sarcomere assembly and function may explain the compensatory responses illustrated by the TGFβ-signaling and ECM organization-related gene networks described here. Mitral regurgitation secondary to myocardial dysfunction is well known in human patients (Asgar et al. 2015). Interestingly, myocardial fibers are present in the proximal third of the mitral valve in dogs (Buchanan 1977; Fox 2012). Thus, it is possible that the changes in valvular gene expression, relevant for myocardial function, affect valvular performance and integrity. The observed degree of differential expression as well as the known function of *MYH1* and *CNTN3* may suggest that genes encoding heart muscle proteins play a role in MMVD and CHF. However, further investigations are needed to clarify this.

### Supplementary Information

Below is the link to the electronic supplementary material.

Supplementary file1 (XLSX 30 KB)

Supplementary file2 (XLSX 50 KB)

## Data Availability

All data are made available in the Gene Expression Omnibus repository. GEO accession: GSE217750.
